# Supplementary material for: Conditions for replay of neuronal assemblies
Source: PLoS Comput Biol. 2026 Jan 16;22(1):e1013844. doi: 10.1371/journal.pcbi.1013844 (PMC12829973; doi:10.1371/journal.pcbi.1013844)
Supplement: S1 Appendix — We perform a quantitative comparison between the pulse width and speed of successful replays, measured in the simulations shown in Fig 1–D and Fig 4–A,C. (PDF) [file pcbi.1013844.s001.pdf]

S1 Appendix Relation between pulse width and speed

For successful sequence retrieval, we measured the pulse speed and width, both in the spiking network and population model simulations. The pulse speed measures how fast the pulse travels from one assembly to the next, thus telling us how long the sequence will take to be replayed. On the other hand, the pulse width, given by the full width at half maximum (FWHM) of an assembly's population rate, is proportional to how long it takes for an assembly to fully cross the threshold. As we saw in our spiking simulations, when feedforward connectivity is very strong, recurrent connections are not needed for retrieval, with propagating pulses being fast and narrow. Conversely, when feedforward connectivity is weak, recurrent connections are needed for retrieval, with pulses being slower to propagate and having a larger width. Thus, we expect pulse with and speed to be correlated, as suggested by Fig 1–D. Below, we quantitatively compare the width and speed of activity pulses in the simulations of Fig 1 and Fig 4–A,C. Our analysis shows that both pulse width and speed are clearly correlated across all models.

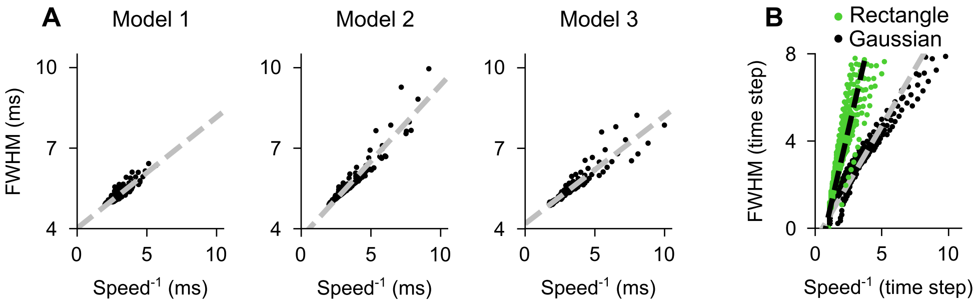

Fig S1.1. Correlation between pulse width and speed. **A**, Asymptotic pulse speed and width (FWHM) of the three spiking models in Fig 1–D1:D3. The Pearson correlation coefficients are: 0.916 for the first model, 0.958 for the second model, and 0.945 for the third model (p-value < 0.001 for all three). The dashed line corresponds to the best-fit linear regression. **B**, Same as panel A, but for the population model (values shown in Fig 4–A,C), with the clipped-Gaussian (black dots) and rectangle distributions (green dots). For better visibility, we limit the analysis to pulses with a FWHM no wider than 8 time steps. The Pearson correlation coefficients are: 0.938 for the clipped Gaussian, and 0.921 for the rectangle (p-values < 0.001 for both cases). The dashed line corresponds to the best-fit linear regression.
