# Supplementary material for: Conditions for replay of neuronal assemblies
Source: PLoS Comput Biol. 2026 Jan 16;22(1):e1013844. doi: 10.1371/journal.pcbi.1013844 (PMC12829973; doi:10.1371/journal.pcbi.1013844)
Supplement: S2 Appendix — We simulate the ‘Model 2’ spiking network (sketched in Fig 1–A2) with different EI ratios and different times for balancing the network. (PDF) [file pcbi.1013844.s002.pdf]

S2 Appendix Spiking models with different excitation-inhibition ratios

In our spiking networks Model 1 and Model 2 (sketches in Fig 1–A1,A2), there are $N_E = 20,000$ excitatory cells and $N_I = 5,000$ inhibitory cells, which corresponds to an excitation-inhibition (EI) ratio of 4:1. Here, we show what happens to the results shown in Fig 1 when this ratio is changed. For simplicity, we use Model 2 and change the EI ratio by changing the number of inhibitory neurons N_I.

For simulation results to be comparable when N_I is changed, we scale connection probabilities or the strength of individual synapses (for a list of parameter values and details on how they are changed with respect to the simulations in Fig 1, see Table S2.1 and 'Scaling the synapses' below). We then perform the same simulation protocol used in Fig 1. The results obtained when the EI ratios are 2:1 and 8:1 are shown below:

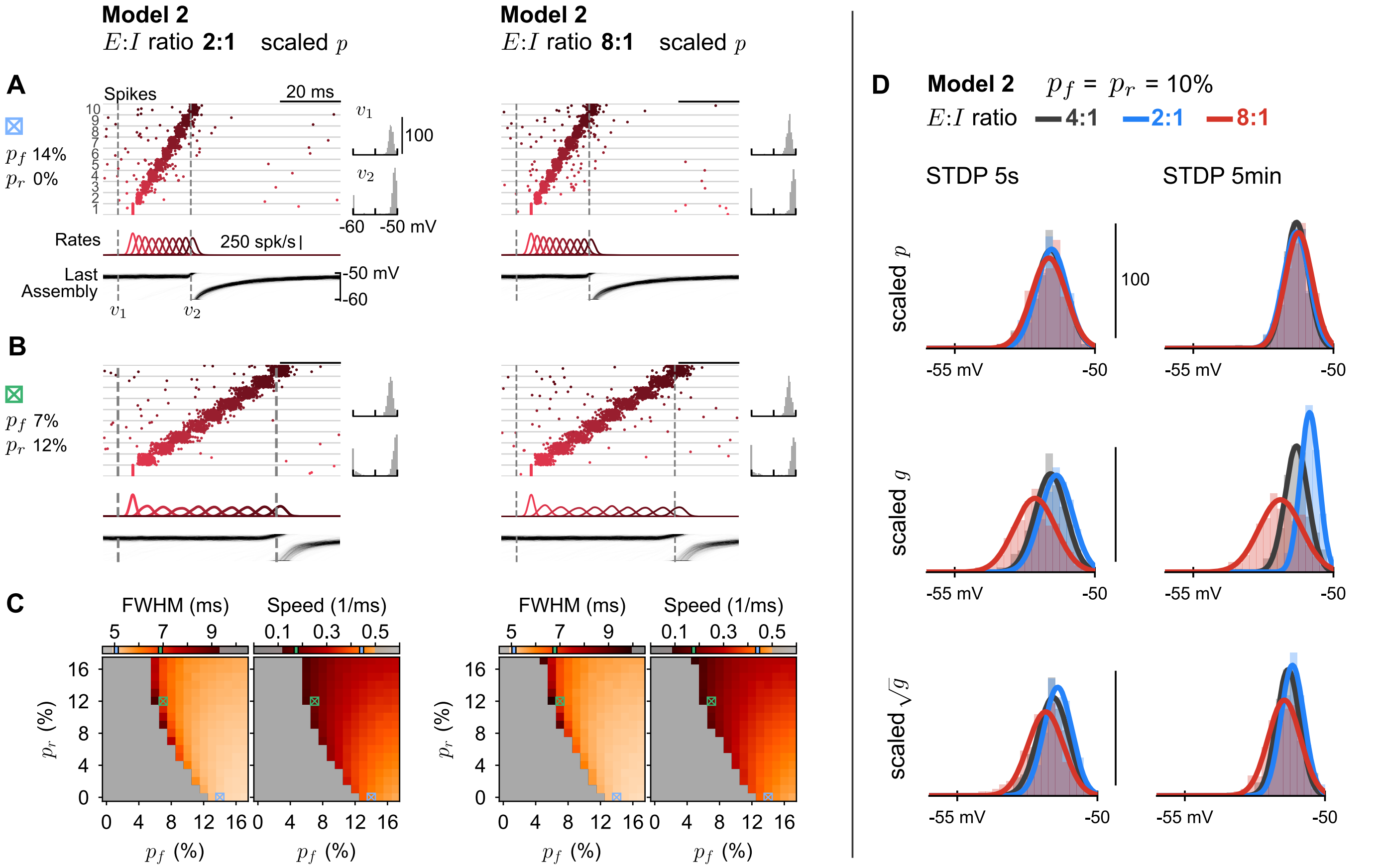

Fig S2.1. A–C, Sequential activation of neuronal assemblies with different EI ratios. Same as Fig 1, but we simulate Model 2 with EI ratios 2:1 (left) and 8:1 (right). This is obtained by doubling or halving the number of inhibitory neurons N_I in the model, respectively. To maintain synaptic strengths as in Fig 1, we scale the connection probabilities p_{ei} and p_{ii}. **D, Membrane potential distributions of the last assembly in the sequence.** Shown for EI ratio 4:1 (gray), as in Fig 1, and compared to ratios 2:1 (blue) and 8:1 (red). Three different synaptic scaling methods are used (see 'Scaling the synapses' below). In the first row, we keep the synaptic input constant by scaling the connection probabilities ('scaled p'), as in panels A–C. In the second row, we scale the weight of the individual synapses to keep the mean input constant ('scaled g'). Finally, in the third row, we scale the weights to keep the noise of the synaptic input constant ('scaled $\sqrt{g}$'). The distributions are plotted after running the I-to-E STDP mechanism for 5 seconds (left), as in Fig 1, and for a longer period of 5 minutes (right). Overlain are solid lines corresponding to the best Gaussian fit for each distribution. Table S2.1 lists the parameter changes with respect to the simulations of Fig 1.

The two cases shown in panels A–C (2:1 and 8:1 ratios) are qualitatively and quantitatively very similar to each other and to those of Fig 1, where a 4:1 ratio was used. Thus, the EI ratio used in the main text is not essential to reproduce the dynamics being investigated. However, as predicted by our theory, these results can only be quantitatively similar if the membrane potential distributions of the sequence are equivalent. For this to occur, both the mean and the noise of the synaptic input received by the sequence must remain unchanged. In the simulations

shown in panels A–C, this was achieved by scaling connection probabilities (see ‘Scaling the synapses’ below for details). Panel D shows that this scaling (top row, ‘scaled p’) makes the membrane potential distributions practically indistinguishable regardless of the EI ratio used. This behavior was expected because the number of synaptic inputs per neuron ($N \cdot p$) was kept constant.

As the I-to-E synapses in our spiking networks are plastic, it is not trivial to predict what happens to the distributions if the synaptic weights are scaled instead. In panel D, we show what happens if synaptic weights are scaled to keep the mean or noise of the synaptic input constant before learning (‘scaled g’ and ‘scaled $\sqrt{g}$’, respectively). In those cases, the membrane potential distributions after learning are different for the different EI ratios (even if the training times are much larger than those used for the first scaling method). Thus, scaling the strength of individual synapses leads to different replay dynamics in spiking simulations. Investigating which scaling laws or plasticity mechanisms are used by neuronal networks is beyond the scope of our work.

Scaling the synapses

As we change the EI ratio by changing the number of inhibitory neurons N_I, the synaptic input received by each population also changes. The mean inhibitory input current a neuron receives is proportional to

$$N_I \cdot p_{EI} \cdot g_{EI} \quad \text{(for an excitatory neuron)}$$
$$N_I \cdot p_{II} \cdot g_{II} \quad \text{(for an inhibitory neuron)}$$

where p_{JI} is the connection probability and g_{JI} is the strength of an individual synapse between a neuron in the inhibitory population I and a neuron in a postsynaptic population $J \in \{E, I\}$ (for details, see Methods and Table 1 in the main text). Moreover, the noise of these inhibitory currents is proportional to

$$\sqrt{N_I \cdot p_{EI}} \cdot g_{EI} \quad \text{(for an excitatory neuron)}$$
$$\sqrt{N_I \cdot p_{II}} \cdot g_{II} \quad \text{(for an inhibitory neuron)}$$

To keep these inputs as similar as possible to those in the simulations of Fig 1 when changing N_I, we scale one of the other two network parameters: p_{JI} or g_{JI}. To keep the number of synaptic inputs per neuron constant, when scaling N_I by a factor of γ, we scale p_{JI} by a factor of $1/\gamma$. This keeps both the mean and the noise of the synaptic inputs unchanged. Alternatively, we can scale g_{JI}, which can be done in two different ways: First, to keep the mean input constant, we scale g_{JI} by a factor of $1/\gamma$; second, to keep the noise constant, we scale g_{JI} by a factor of $1/\sqrt{\gamma}$. In Fig S2.1, we refer to each of these three scaling methods as ‘scaled p’, ‘scaled g’, and ‘scaled $\sqrt{g}$’, respectively.

Note that the I-to-E synapses are plastic. Thus, scaling g_{EI} means changing the initial parameter g_0^{EI}. The final synaptic strengths g_{EI} are then learned during training and will differ between neurons. In contrast, all I-to-I synapses have constant strength g_{II} throughout the simulations.

Table S2.1. Parameter changes between Model 2 simulations in Fig 1 and equivalent networks with different EI ratios in Fig S2.1. For each EI ratio, three different synaptic scaling methods are used: named in the table as ‘p’, ‘g’, and ‘$\sqrt{g}$’. Parameters that differ from those used in Fig 1 are in bold.

Parameter		Fig 1 (4:1)	EI ratio 2:1			EI ratio 8:1			Definition
			p	g	$\sqrt{g}$	p	g	$\sqrt{g}$	
p^{II}	(%)	1.0	**0.5**	1.0	1.0	**2.0**	1.0	1.0	(Global) I-to-I connectivity
p^{EI}	(%)	1.0	**0.5**	1.0	1.0	**2.0**	1.0	1.0	(Global) I-to-E connectivity
g^{II}	(pS)	400	400	**200**	**∼283**	400	**800**	**∼566**	I-to-I synaptic weight
g_0^{EI}	(pS)	400	400	**200**	**∼283**	400	**800**	**∼566**	I-to-E initial synaptic weight
N_I		5,000	10,000			2,500			Number of inhibitory (I) cells
