# Supplementary material for: Conditions for replay of neuronal assemblies
Source: PLoS Comput Biol. 2026 Jan 16;22(1):e1013844. doi: 10.1371/journal.pcbi.1013844 (PMC12829973; doi:10.1371/journal.pcbi.1013844)
Supplement: S3 Appendix — We simulate our minimal spiking network (sketched in Fig 1–A3) with excitatory synapses that act as delta-current pulses. (PDF) [file pcbi.1013844.s003.pdf]

# S3 Appendix  Minimal spiking model with instant synapses

In all spiking models simulated in Fig 1 and Fig 2, $E \to E$ synapses were modeled as jumps in conductance that decay exponentially — see Eq (11) and Eq (12) in the Methods. Here, we simulate our third spiking model (sketch in Fig 1–A3) and replace the conductance-based synapses with synapses that act as delta-currents, causing an instantaneous jump $w^{EE}$ in the membrane potential of the postsynaptic neuron after a delay $\tau_l^E$. We perform the same simulation protocol used in Fig 1. The results are shown below:

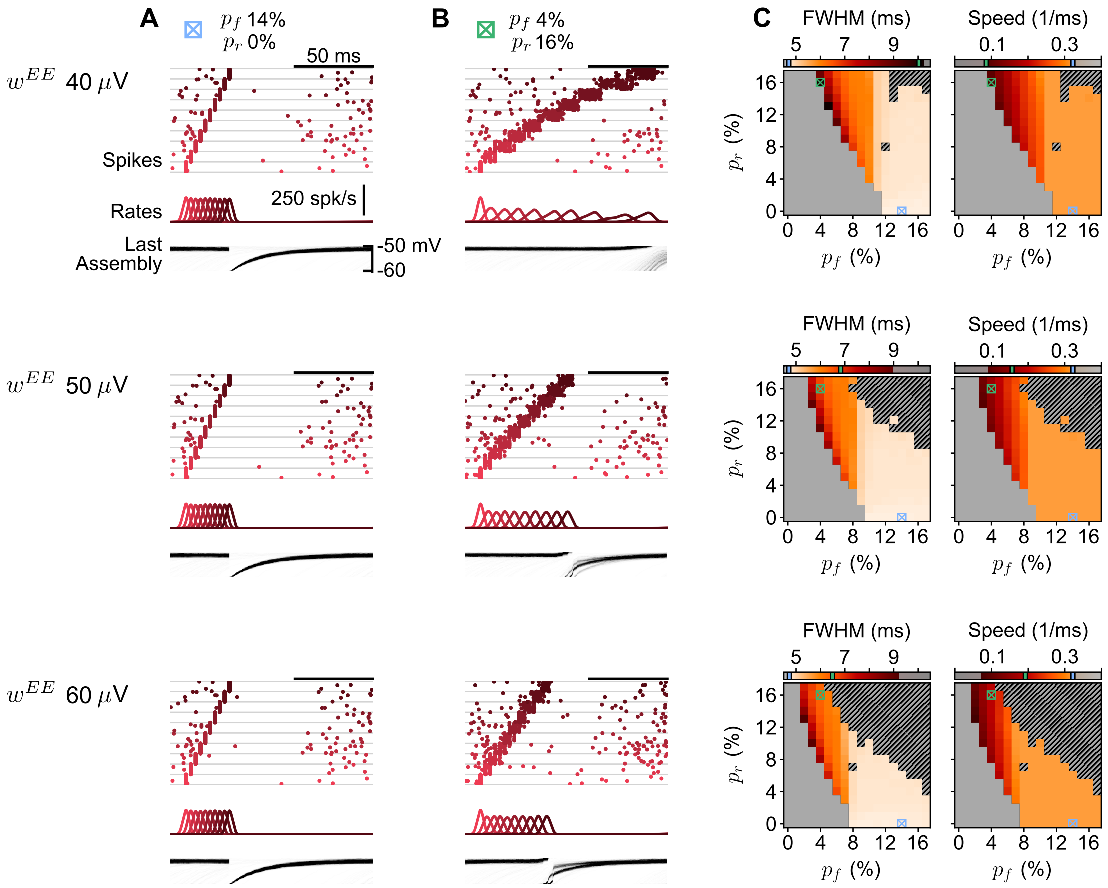


**Fig S3.1.** Same as Fig 1, but we simulate Model 3 with delta-current synapses with synaptic delay $\tau_l^E = 3$ ms and three different synaptic weights $w^{EE}$.

These results show that the choice of synaptic model used in the main text is not essential to reproduce the dynamics being investigated. Moreover, we show that strengthening or weakening the weight of excitatory synapses $w^{EE}$ moves the regions where replay can succeed, as expected (see Eq (17) in the main text).

The synaptic currents that a leaky integrate-and-fire (LIF) neuron $j$ receives are now given by

$$I_j^{\text{syn}}(t) = C \sum_{e,f} w^{EE} \delta(t - t_e^{(f)} - \tau_l^E) + C \sum_{p,f} w^{EP} \delta(t - t_p^{(f)} - \tau_l) \qquad \text{(S3.1)}$$

with $\delta$ being the Dirac delta function, $t_e^{(f)}$ the $f$-th incoming spike from excitatory neuron $e$, $t_p^{(f)}$ the $f$-th incoming spike from the Poisson unit $p$, and $\tau_l$ the latency between a presynaptic spike in a Poisson unit and the postsynaptic response. The quantity $w^{EE}$ denotes the unitary membrane potential increase resulting from a single spike in the excitatory population $E$, whereas $w^{EP}$ is the unitary increase resulting from a single spike in the Poisson population $P$. In our simulations in the main text, we used conductance-based synapses with $\tau_l = 1$ ms and a conductance exponential decay of 2 ms. As our neurons have time constant $\tau_m = 20$ ms, the average point in time at which the postsynaptic potential increases was ∼3 ms after the presynaptic neuron spikes. To approximate this, here we chose $\tau_l^E = 3$ ms.
