# Supplementary material for: Conditions for replay of neuronal assemblies
Source: PLoS Comput Biol. 2026 Jan 16;22(1):e1013844. doi: 10.1371/journal.pcbi.1013844 (PMC12829973; doi:10.1371/journal.pcbi.1013844)
Supplement: Attachment — Submitted filename: Response to Reviewers.pdf [file pcbi.1013844.s004.pdf]

Response to Reviewers

Editor

The reviewers generally appreciate the work presented in the manuscript. However, addressing certain concerns raised by the reviewers could further improve the clarity and overall quality of the paper.

To address major concerns by reviewers #1 and #3, we have significantly restructured the order in which arguments are presented in the manuscript:

- The Results now contain 4 subsections:
    - First, “Sequence retrieval in spiking networks”, where we describe how recurrent connections facilitate replay across three different model architectures, introducing a minimal model with E-only assemblies. This has stayed mostly unchanged since the initial submission.
    - Second, “Minimal spiking model reveals key replay mechanisms”, where we simulate variations of the minimal spiking model and show that synaptic speed and membrane potential distribution are key to determining replay success. In our original submission, this was only shown at the end of the results, after the population model.
    - Third, “A time-discrete population model for replay”, where we introduce our population model and show with numerical simulations that its behavior is qualitatively similar to that of the spiking networks.
    - Fourth, “Population model predicts analytical conditions for replay”, where we use our population model to derive analytical predictions for replay success and speed, and show that these match spiking network behavior. In the original submission, the third and fourth sections were presented together and between spiking network results. Many analytical derivations are now exclusively in the Methods to facilitate reading and to better highlight the importance of our findings.
- A previous appendix with analytical derivations for the population model is now embedded within the Methods in the main text.

We believe the manuscript now presents our findings in a much more cohesive and clear way, and we hope this addresses the reviewers’ main issues with our original submission.

Below, we provide a point-by-point response to each of the reviewers’ comments.

Reviewer #1

In this paper, the authors investigate the fundamental conditions for groups of neurons to fire simultaneously in precise sequences, known as synfire chains. In particular, the authors rather than emphasizing the significant role of inhibitory feedback, demonstrated and highlighted the critical importance of the interply between inter-assembly feedforward connectivity and the intra-assembly recurrent connectivity. These structural motifs (at multiple scales and hierarchies) are shown to be key factors for amplifying and sustaining neuronal synfire chain propagation.
To support their observations and conclusions, the authors moreover developed an analytical framework meanwhile constructed minimal SNN models. Their combined analytical and numerical results convincingly demonstrated that recurrent connections alone can support the generation and replay of sequential activity.
This work contributes to our understanding of the mechanisms underlying synfire chains and sequence replay, with potential implications for theoreis of memory consolidation and sequential processing.

Overall, I think this work to be of high relevance and suitable for Plos CB, however, several major revisions and clarifications are necessary before it can be considered for publication.

Many thanks for the encouraging summary and feedback.

Major comments

1. The paper appears to be organized into three main parts: it begins with a behavioral analysis and comparison of sequence retrieval across different SNN architectures, then introduces a theoretical population model to quantify key aspects of sequence retrieval and identify three essential conditions for successful replay, and finally returns to the minimal spiking models to validate the theoretical predictions through numerical simulations.
In the first part, such as Fig. 1 and related results, the authors seem to focus on the role of E-I balance by comparing three SNN architectures, then demonstrating (emphasizing?) that inhibitory feedback is not a necessary condition for sequence replay. In the second part, however, the theoretical framework seems to be developed specifically based on the minimal SNN (Model 3), which only includes excitatory neurons. Here, the emphasis shifts from inhibition to the interaction between feedforward (F) and recurrent (R) connectivity. This analysis leads to the identification of three critical conditions for sequence replay, formulated as functions of F and R. In the final section, the study returns to the minimal SNN to numerically validate the influence of F-R interaction on sequence replay.
This article flow is good, but I believe a reorganization could improve the clarity and narrative logic of the manuscript. For instance, the authors might consider restructuring the presentation to first introduce the three SNN architectures to show that inhibitory feedback is not essential. Then, focusing solely on the minimal model (model three), they could deeply explore how varying network parameters affects sequence retrieval, as done in current Fig. 4. This exploration would naturally motivate the development of the theoretical framework, which would then serve to explain and generalize the key observations, culminating in the derivation of the three critical conditions for successful replay.
Of course, the authors may have an alternative structure in mind that best fits their goals, and I leave the decision to them.

Thank you for the detailed suggestion in restructuring the paper, with which we fully agree. We have performed a major restructuring of our Results section. Please see also our response to the comments by the Editor above for a detailed summary of our changes.

I would moreover recommend that the author put less emphasis on the unnecessary of the inhibitory feedback.

Our focus on this finding was motivated by the fact that the only model that had so far (to our knowledge) shown that Hebbian assemblies can facilitate feedforward propagation of pulses was that of Chenkov et al. (2017), which hypothesized the necessity of a balanced EI state. However, our simulations of Fig. 1 do show that inhibitory feedback, although not needed, plays a significant role in modulating the properties of feedforward pulses, which we do not explore in this manuscript. We therefore had put a lot of emphasis on inhibitory feedback. However, we agree with the reviewer's suggestion that this emphasis should be reduced because our main finding is that excitation is more important. Thus, we have removed the previous Discussion subsection titled "Hebbian assemblies can amplify activity pulses without inhibitory feedback" and merged it into a single subsection on the role of Hebbian assemblies in sequence replay. Moreover, we no longer mention this particular finding in our abstract or when summarizing our findings in the introduction.

2. The limitation of the current population model appears to be missing from being discussed. For example, if I understand correctly, the model resembles a rate-based or mean-field approximation, relying on the assumption that individual neurons within an assembly activate independently. However, this assumption may break down when recurrent excitation (e.g., W^{EE}) becomes sufficiently strong. In such regimes, the activation of a single neuron could trigger a cascade of activity within the assembly, resulting in a so-called "neural avalanche"
(see for example https://journals.aps.org/prl/abstract/10.1103/PhysRevLett.134.028401).
This phenomenon may correspond to the "explosion" described in the manuscript.
I understood that the authors have taken care to embed their analysis within an appropriate parameter regime, it would be better to explicitly state the limitations of the population model and clarify the valid operating regime in which its assumptions hold.

We agree that our initial submission was not sufficiently clear in addressing the limitations of our models. To correct this, we've added more detail to the subsection "Model assumptions" when introducing the population model, and created a whole subsection in the Discussion titled "Main assumptions and limitations of our replay models", where we summarize our key assumptions and resulting limitations.

The reviewer is correct in pointing out that, for our mean-field approximation to work, we need EPSPs (w^{EE} in our model) to be small enough. To make this clear, we've added the following to our Discussion (line 651):

> *"Our population model (Fig 3 and Fig 4) assumes that a mean-field approximation of the dynamics of the spiking model is possible. Then we can define the strength of recurrent and feedforward connections as the product of the number of assembly neurons M, the connectivity p, and the postsynaptic potential w^{EE}; see Eq (3). For this*

approximation to hold, we require a sufficient number of synapses within and across assemblies ($M\,p \gg 1$) and that individual postsynaptic potentials w^{EE} are not too large."

Another limitation we now explicitly address is that our population model cannot describe the activity cascades mentioned by the reviewer. These require each neuron to fire more than once. For simplicity, we choose to focus on the cases where neurons only fire once, on average, which are the focus of synfire chain models, for example. Thus, we neglect what happens to neurons after they reset. To make this even clearer, we've now added the following sentence to the "Model assumptions" paragraph (line 334):

"From this assumption, it follows that the population model can never predict when activity explosions or bursting may occur."

And in the Discussion, we say (line 658):

"We further assume that in a successful replay event, sequence neurons need only fire once, on average. This allows us to neglect reset dynamics in our population model, which will therefore not be able to predict when activity explosions or bursts occur (see the top-right corner in Fig 1-D3). This means that our population model is only valid in a regime where runaway excitation is impossible, i.e., we are always in a subcritical regime where avalanches such as those studied in Yang et al. (2025) cannot occur."

And the authors state that the population model provides a better approximation as the sequence length q increases. A related question is whether the model also implicitly assumes a sufficiently large number of neurons within each assembly? I would expect that having a large enough assembly size is necessary to ensure that the statistical assumptions of the voltage distribution remain valid.

We agree with the reviewer and have now added the following text in our Discussion (line 683):

"Our population model further assumes that an increase in the mean of an assembly's membrane potential distribution will generate activity in the network, proportional to the area of the distribution that crosses the firing threshold (see sketches in Fig 3). This assumption requires that there is a sufficient number M of neurons per assembly, such that the distribution is well populated across the entire width U. Thus, our results are valid only if $M \gg 1$."

3. The authors emphasize that recurrent and feedforward connectivity work in concert to amplify neuronal activity and facilitate sequence retrieval. There is also a complementary line of research that explores the competition between these two forms of connectivity. These studies highlight their respective roles in balancing and regulating information transmission versus information processing and computation, for example, Clark, D. G., & Beiran, M. (2025) PNAS; Wang, Z., Sornborger, A. T., & Tao, L. (2016). PlosCB. It would strengthen the manuscript if the authors could briefly discuss this perspective in the discussion section, particularly in the broader context of how recurrent and feedforward pathways jointly shape information flow in the brain.

Thank you for pointing us to these studies, which help contextualize our work within the existing literature.

We now mention Clark and Beiran (2025) when discussing the role of Hebbian assemblies in sequence replay (line 733):

> *"Interestingly, a recent theoretical study (Clark and Beiran, 2025), investigating how activity propagates throughout the brain, found that within-region and cross-region connectivity can play competing roles in the generation and transmission of neural signals."*

And we mention Wang et al. (2016) when discussing how the interplay between recurrent and feedforward connections shapes the properties of the propagating activity pulse (line 811):

> *"Interestingly, Wang et al. (2016) found that, in a purely feedforward model, it is possible to decrease the synchrony of activity pulses in synfire chains by having a second sequence regulate the activity of the first one. Our findings suggest recurrent connections among Hebbian assemblies can provide a different mechanism by which the transfer of wide (less synchronous) activity pulses can occur across neuronal circuits."*

Minor comments

1. Some subsubsections could be merged for improved coherence and flow. For example, the sections "Relation between pulse speed and connectivity" and "Necessary connectivity given a minimum speed" both address the relationship between sequence speed and connectivity structure and could be merged.

This and other such simplifications have been done in the new restructured version of the manuscript.

2. The line following S3. 22, a typo "with".

This text is no longer present in the new version of the manuscript.

Is the second line in S3.58 missing a (q-1)/(mq-1) term?

Many thanks for pointing this out to us. We've now changed the text to explicitly mention that this term is also included in our fraction of polynomials. Note that in the new restructured version, this is now Eq. (87) in the Methods.

Reviewer #2

The paper examines neuronal sequences through synfire chains to simulate hippocampal replay. Interestingly, it finds that feedback inhibition is not required for the formation of firing sequences. Additionally, the paper provides an analytical description of how various connectivity structures influence speed, indicating that weaker feedforward connections produce slower pulses, which can be maintained by recurrent connections. Below are several suggestions and queries that we hope will strengthen the paper.

Major comments

1. (Major) The model establishes a fixed ratio of E- to I- cells at 4:1. However, Gandolfi et al. (2023) reconstructed the human CA1 region using a 9:1 cell-type ratio. Would an E:I ratio greater than 4 influence your key analysis on "conditions for replay" or the stability of sequence propagation? If so, please discuss the sensitivity of the parameters involved and the physiological rationale for selecting a 4:1 ratio.
Reference: Gandolfi, D., Mapelli, J., Solinas, S.M.G. et al. Full-scale scaffold model of the human hippocampus CA1 area. Nat Comput Sci 3, 264–276 (2023).
https://doi.org/10.1038/s43588-023-00417-2

The strength of the E-I connections in our spiking networks depends not only on the number of cells in each population but also on the synaptic properties. In models 1 and 2, we used plasticity in the I to E connections (as in Vogels et al. 2011) to reach our desired low-firing asynchronous-irregular state. Thus, many different parameters could have been chosen to achieve this, and we could also have chosen a different cell ratio. Our choice of a 4:1 ratio was done only for simplicity, as it was the standard choice in computational models on which we base our work, e.g., Chenkov et al. (2017), Vogels et al. (2011). As we do not focus on the specifics of how inhibitory currents modulate sequence replay, we remain agnostic about these properties. Moreover, we believe our models are general enough to apply not only to the hippocampal areas CA1 and CA3, but also to neocortical areas where sequential activity is observed. To make this clear, we now explicitly state this when introducing cell numbers in the Methods (line 937):

> *"Note that the 4:1 excitation-inhibition ratio is chosen for simplicity and is not critical to reproduce our results. Different ratios have been reported across brain regions (see, for example, Gandolfi et al., 2023)."*

To make clear what parameters we believe are crucial to reproduce our results, we now discuss more explicitly what assumptions are key to our model throughout the text, including in a new Discussion subsection titled "Main assumptions and limitations of our replay models".

2. (Major) The model fixes the number of excitatory assemblies at q = 10 for insets A and C of Figure 3. How about other numbers of assemblies, say, q = 200?

In our spiking networks, we chose $q=10$ to ensure efficient simulation times. Exploratory simulations with much larger values of *q* showed that 10 assemblies appear to be long enough to approximately capture the asymptotic properties of sequential activity pulses, and to test

whether these could be indefinitely sustained. This means that, if we observed replay succeeds down to the 10th assembly, then it would also approximately succeed down to the very last one, regardless of how long a sequence is. The same was observed by Chenkov et. al (2017). We also mention this in the Results (line 60):

> *"In each network, we embed a sequence of 10 assemblies. This number is chosen because it is short enough to allow for quick simulation times, but long enough to approximate the asymptotic pulse behavior in very long sequences."*

The concern of keeping an efficient simulation time does not apply to our population model, whose numerical simulations we showed in Fig. 4 (previously numbered Fig. 3). There, in panels A-D, we chose $q{=}10$ merely to ease the comparison with the spiking simulations we aim to describe. We then show with the numerical simulations of panels E-F that the population model predicts qualitatively the same behavior for any sequence length (we show up to $q{=}500$). Moreover, we show that our analytical Condition 3, although a decent approximation for $q{=}10$, is a theoretical limit of the rectangle model as $q{\to}\infty$, becoming a better match the longer the sequence is.

3. (Major) The current model applies STDP mechanisms only at I to E synapses to homeostatically regulate EI balance without altering the fixed feedforward chain. It would be valuable to discuss what happens if STDP were also applied to E-E, E-I, or I-I connections where these STDPs have existed in real animal experiments. For example, E-E plasticity might enable the assembly chain to be learned or refined, but could drift away from the derived replay conditions unless counterbalanced by homeostatic mechanisms. Similarly, plasticity at E-I or I-I synapses could modify pulse width and propagation speed. A brief discussion or supplemental simulations exploring these scenarios would clarify the robustness of the replay conditions.

We agree that plasticity across different connections should have a strong effect on the formation and retrieval of assembly sequences, particularly in the connections within excitatory cells. Due to the immense complexity of the topic, on which countless experimental and theoretical studies have focused, we have chosen instead to focus in the Results only on the dynamics of a single retrieval of a manually embedded sequence, remaining agnostic about plasticity rules. Nevertheless, it would be important to clarify the putative impact of plasticity at other synapses. Therefore, we restructured our Discussion such that there is a subsection about this topic (see "Replay and plasticity of synaptic connections"), with several references to the literature.

We believe that a more detailed investigation of the plasticity among E-I and I-I connections falls outside the main scope of our study. The strength of our work is in showing the key ingredients that allow our minimal spiking network (without inhibition) to replay a sequence, which can be well approximated by our new population model. Thus, we have chosen not to study the role of inhibitory connections during replay. We now also state this as a clear limitation of our model in the Discussion subsection "Main assumptions and limitations of our replay models":

4. (Major) I wonder about the situation if there is a model 4 that has background connections but only uses Poisson neurons as inhibitory assemblies?

We think such a configuration would require additional mechanisms like synaptic depression of neuronal adaptation to prevent runaway excitation, since a large excitatory network without any feedback inhibition would easily "explode" (such cases are discussed in the last paragraph of our Discussion). One of our key findings is that feedback inhibition is not needed for amplification within an assembly during replay. However, we believe that feedback inhibition is likely needed to maintain a baseline low-firing state in large neuronal networks.

5. (Major) In lines 100-103, the authors discuss activity pulse propagation. I wonder if there is empirical criteria or specific firing-pattern benchmarks (e.g., propagation delay, spike-time synchrony) they would use to determine that the modeled propagation accurately reflects real anatomical data.

To our knowledge, it is currently impossible to compare activity pulses between our models and real data. We have chosen to analyse the role of connectivity within and across neuronal assemblies and to evaluate propagation beyond simple failure or success by measuring pulse properties at each assembly in the sequence. We have chosen these measures although, with current experimental methods, one cannot quantify this in data. For replay during hippocampal sharp wave-ripples, for example, where a replay event (the whole sequence) has a duration of about 20-50 ms (as in our simulations where q=10), it has not been possible to separate assemblies and to define where the activities of assemblies start and end.

6. (Major) In lines 146–147 the authors identify p_f = 0.12 as a threshold for stable propagation. Please clarify whether this critical feed-forward probability depends on network scale—specifically total excitatory neuron count (N_E) or assembly number (q)—and if so, how p_f shifts when these parameters vary.

We think the structure of our initial submission led to a key finding being lost within the details: the 12% threshold was a result of our (non-special) parameter choice. We now make our findings of Fig 2-B (old Fig 4-B) much more central to the manuscript and discuss them in detail: varying the membrane potential distributions in the AI state significantly changes where this threshold is located. Note that our population model predicts that this threshold will be approximately located at $F = U - x_0$. For $x_0 = 0$, we find the simple relation $p_f \cong U \,/\, (M\, w^{EE})$.

7. (Major) The manuscript does not explain why it chose N_E = 5000 in model 3 but 20000 in models 1 and 2.

There are no background neurons in Model 3. Because there we chose $p_{bg} = 0$, we effectively are left with $N_E = q\ M = 5{,}000$, i.e., only the sequence neurons exist. On the other hand, models 1 and 2 had 15,000 "background" excitatory neurons that did not make up the sequence but could connect to sequence neurons with a low probability p_{bg} > 0. We now make this clear in our Results section (line 192):

"The third spiking model (Fig 1-A3) is the simplest that can exhibit replay of assembly sequences. It consists merely of the sequence of excitatory assemblies and has neither

inhibitory neurons nor background connections ($p_{bg} = 0$). This setup effectively disconnects the sequence from the background neurons, and we can consider a smaller network size $N_E = q\,M = 5{,}000$."

8. (Major) In Equation 19, the analysis focuses solely on the W_EE. Please clarify why other synaptic weights (e.g., W_EI, W_IE, W_II) were not included in the propagation criteria. How would varying those other weights affect the derived conditions for replay and the stability of sequence propagation?

The analytical insights derived from the population model are an approximation of the dynamics of our minimal spiking network (Model 3), which only contains excitatory cells. It would be interesting to consider how the E-I connections modulate our findings, but this falls outside the scope of the work we presented. However, our model predicts that the properties of the subthreshold membrane potential distribution affect the replay, and this distribution is shaped by various weights in the network, including connections to and from interneurons. We now make sure to clearly state that this is a limitation of our work, by saying in the Discussion (line 702):

"Although it is important to study these inhibitory effects, they are not captured by the population model introduced here, which, for simplicity, we have kept as simple as possible. Future work could focus on whether our new theoretical approach may also describe the effect of inhibitory currents during replay."

Minor comments

9. (Minor) The manuscript contains many parameters, which the authors have organized into Table 1. However, this arrangement may make it difficult for readers to easily reference. They need go back and forth to read this Table. I recommend that the authors include the specific value for each parameter immediately for improved readability. This change would maintain the table's formal structure while enhancing clarity.

We agree that the main text was too vague on this, so for clarity we have now added explicit mention to constant parameter values like N, M, and p_{bg} in the Results (text and Fig 1 caption) and Methods. However, most parameter values are still not mentioned explicitly. This choice was motivated by the fact that many different parameter configurations could be chosen to achieve what is shown. We can arrive at approximate analytical descriptions of the spiking network behavior with our new population model approach, which contains the parameters we believe to be most significant: effective connection strengths R and F, and distribution width U. Thus, we believe the actual parameter values used in spiking simulations are most important only to readers who might wish to reproduce our results or go deeper into the spiking dynamics. Moreover, other reviewers showed concern that our Results section was too muddled with information that was not essential to our key findings, which motivated us to remove many analytical derivations to the Methods. For these reasons, we have kept many spiking network parameter values exclusively in Table 1, as in the original submission.

10. (Minor) For further clarity, each horizontal row in Figure 1B and 1C corresponds to one assembly (IDs 1-10) with the bottom-to-top rows, representing Assembly 1, the new row Assembly 2, and so on.

We now indicate assembly indices 1-10 in the raster plot in panel B1.

11. (Minor) The Figure 1 inset A3 missed a "N_E" beside the N_E assemblies.

12. (Minor) Figure 2 has unlabeled x-axes and missing arrows on the x-axes.

Thank you for spotting these errors; they are now fixed.

13. (Minor) The notation g_t(v) (Eq. 1) for the membrane-potential distribution may be mistaken for conductance. Consider using other notation to avoid confusion.

We agree and have now replaced g by z to avoid confusion. Note that this is now in Eq. (22).

Moreover, h_U(v) is a normalized function (in line 286), while it is a rectangle model in line 326. Please consider different notations as well.

Our model states that h_U can be any normalized function. The rectangle model is the instance where that function h_U is a rectangle. Therefore, we think it is best to keep the same notation in this case. To make this clear, we now introduce the function in the following way (line 1096):

> *"The static shape has finite width U and is described by a normalized function h_U [...]. In general, the function $h_U(v)$ can have any shape as long as these conditions are met."*

14. (Minor) In Figure 3 panels A and C, please specify the heat maps plot F and R values in units.

In the population model, F and R are adimensional, with the relevant scale always being that of the distribution width U. For simplicity, we chose $U=1$, such that F or R being equal to 1 corresponds to them being equal to the distribution width. Note that, due to the restructuring done for this new submission, this figure is now numbered Fig 4.

15. (Suggestion) Please verify whether some corrections will affect the results.

We have rechecked everything and are confident that the discrepancies/errors found by reviewers do not affect our results.

Reviewer #3

This manuscript investigates how recurrent connections can support the replay of sequential neural activity in LIF spiking networks, even in the absence of inhibition or balanced amplification. The authors use a combination of simulations and a time-discrete population model to characterize conditions under which sequence retrieval (or replay) occurs and to quantify how replay speed and pulse width depend on network connectivity parameters. The study provides mechanistic insight into how excitatory networks alone can generate propagating activity patterns, a scenario relevant to cortical and hippocampal function. The manuscript addresses an important question in theoretical neuroscience and proposes a simplified yet analytically tractable framework. The findings challenge the conventional emphasis on inhibitory mechanisms in sequence replay, offering a conceptually original perspective. The use of a population-level membrane potential distribution model is a notable methodological contribution.

Many thanks for the encouraging summary of our work.

However, the manuscript suffers from issues in organization, clarity, and presentation. The argumentation is fragmented, the narrative lacks coherence, and key results are often buried in a sea of technical detail and assumptions. A thorough reorganization is needed to improve readability and highlight the main scientific contributions.

We agree that the paper needed a significant restructuring, which we have now done. Please see our first response to the editor above for a detailed summary of our changes.

Strengths:
1. Introduces a tractable population model using membrane potential distributions.
2. Derives analytical conditions predicting replay feasibility and speed.
3. Provides a mechanistic link between subthreshold membrane potential distributions and replay dynamics.
4. Proposes a minimal model that may better reflect local hippocampal/cortical microcircuits than previously assumed balanced EI models.
5. Exploration of pulse dynamics (speed, width) as functions of connectivity and synaptic time scales.

However, the manuscript is overly dense, with many small technical arguments that obscure the main ideas. The progression from spiking model results to the population model is unclear; better scaffolding is needed to guide the reader.
Model assumptions are central to the population model but are presented in a scattered fashion. Their rationale, implications, and limitations should be further clarified.

Our new restructured version helps to address these concerns. We now have a shorter manuscript with a better focus, with many theoretical details having been moved to the methods. This also highlights the importance of the “Model assumptions” subsection, to which we have added detail. Moreover, we have added a new subsection to the Discussion titled “Main assumptions and limitations of our replay models”, which summarizes the key assumptions used throughout the manuscript.

Equation (1) may be corrected as h_U(v) = g_t^(i) (v-x_t^(i)) in my understanding.

Perhaps the reviewer got misled by our previous ambiguous notation, where we used g to describe both the conductance and the membrane potential distribution. To correct this, we've now replaced g by z to avoid confusion in this context. We also note that this level of detail has been omitted from the Results and is now only present in the Methods -- see Eq. (22).

The main insights (e.g., recurrent excitation alone can support replay; speed–width–connectivity tradeoffs) are not clearly foregrounded.

We hope the new version is much better at conveying our main findings to the reader.

The claim that leak currents are essential in determining subthreshold distribution is made but not explicitly supported by results — adding a dedicated figure or example could help substantiate this.

To address this, we now support our claim in the paper by Brunel (2000), in the "Model assumptions" paragraph when first introducing the population model (line 299):

> *"Leak currents have been shown to be essential in determining subthreshold membrane potential distributions in spiking networks with AI firing (Brunel, 2000), which is the baseline state on which we test replay in our simulations of Fig. 1 and Fig. 2."*

Reference: https://doi.org/10.1023/A:1008925309027

Highlighting the transition from realistic spiking behavior to idealized population-level dynamics would clarify the model's scope.

We believe the new details on assumptions and limitations added (particularly the new Discussion subsection, focused on the main assumptions and limitations of our models) now make clear under which conditions our models are valid.
