# Supplementary material for: Conditions for replay of neuronal assemblies
Source: PLoS Comput Biol. 2026 Jan 16;22(1):e1013844. doi: 10.1371/journal.pcbi.1013844 (PMC12829973; doi:10.1371/journal.pcbi.1013844)
Supplement: Attachment — Submitted filename: Review_round2.pdf [file pcbi.1013844.s005.pdf]

Review Comments

I thank the authors for their comprehensive response and the significant restructuring of the manuscript. The flow is improved, and the distinction between the spiking network results and the population model predictions is much clearer. The addition of the "Model assumptions" and "Limitations" sections is also appreciated. However, I have a few remaining points where I believe further clarification or minor validation would strengthen the manuscript's conclusions.

Follow-up on Major Comment 1:
I appreciate the authors' clarification that the 4:1 E/I ratio was selected mainly for simplicity and consistency with standard modeling studies (Vogels et al., 2011; Chenkov et al., 2017), and that inhibitory plasticity can tune the network into a low-firing asynchronous–irregular state across a range of ratios. However, I remain interested in the sensitivity of the replay dynamics to the E/I ratio itself, as different E/I ratios may all yield low-mean, asynchronous–irregular spiking, but still differ substantially in effective inhibitory gain, timing precision of assembly activation, or the robustness of recurrent-excitation–driven propagation. The authors note that they “remain agnostic” about specific inhibitory properties, which makes it unclear how changes in inhibitory properties impact the full E/I network. Although line 937 confirms that the E/I ratio is not critical for reproducing the results, at present this reads more like an assumption rather than a demonstrated finding. To strengthen the paper, it would be helpful to provide additional evidence demonstrating the robustness of the replay conditions across different E/I ratios.

Another follow-up question:
The model predicts a wide range of replay speeds depending on connectivity and synaptic delay. Can the authors relate the predicted speed range more explicitly to experimentally observed replay compression factors (e.g., during sharp-wave ripples versus awake replay)? Could you please indicate which ranges of F and R are consistent with experimentally measured hippocampal connectivity and synaptic strengths, and whether the successful replay region falls within those plausible ranges?
